# Gestational age–associated differences in human umbilical cord mesenchymal stem cells: biological, paracrine, and neuroprotective properties

**DOI:** 10.1186/s13287-026-05242-8

**Published:** 2026-08-12

**Authors:** Xirui Peng, Yuhang Zhang, Yizhuo Li, Yangyang Cao, Lingling Zhang, Ju Wang, Xiaoli Zhang, Yiran Xu, Yu Yang, Jiajia Duan, Yuyang Yue, Juan Song, Changlian Zhu

**Affiliations:** 1 Henan Key Laboratory of Child Brain Injury and Henan International Joint Laboratory for the Prevention of Child Brain Injury, Henan Pediatric Clinical Research Center, Institute of Neuroscience, Third Affiliated Hospital of Zhengzhou University, Zhengzhou, 450052 China; 2 Department of Anesthesiology, Third Affiliated Hospital of Zhengzhou University, Zhengzhou, China; 3 Center for Brain Repair and Rehabilitation, Institute of Neuroscience and Physiology, University of Gothenburg, Gothenburg, 40530 Sweden

**Keywords:** UCMSCs, Preterm infant, Gestational age, Extracellular vesicles, Transcriptomics, Germinal matrix hemorrhage, Neuroprotection

## Abstract

**Background:**

Human umbilical cord mesenchymal stem cells (UCMSCs) have shown considerable therapeutic promise for neonatal brain injury. However, whether gestational age influences UCMSC biological characteristics and therapeutic efficacy remains unclear. This study investigated the potential functional and molecular differences between UCMSCs derived from preterm and full-term infants to inform future autologous and allogeneic cell-based therapies.

**Methods:**

UCMSCs were isolated from full-term and preterm umbilical cords, with preterm samples further classified into uncomplicated preterm (N-P), gestational diabetes exposure (P-GD), and severe preeclampsia exposure (P-SE) groups. MSC identity, viability, proliferation, apoptosis, trilineage differentiation, and extracellular vesicle (EV) characteristics were evaluated. Transcriptomic analysis was performed to compare N-P and full-term (N-F) UCMSCs, followed by RT-PCR validation of key hub genes. Therapeutic efficacy was assessed in a neonatal germinal matrix hemorrhage (GMH) mouse model following intranasal administration of N-F- or N-P-derived UCMSCs, with neurological function and brain injury evaluated by behavioral testing and T2-weighted MRI.

**Results:**

All UCMSCs met standard MSC characterixation criteria. Compared with N-F UCMSCs, N-P UCMSCs showed increased cell viability as assessed by CCK-8, enhanced chondrogenic differentiation potential, and increased secretion of EVs expressing CD9, CD63, and CD81. In contrast, UCMSCs from the P-GD and P-SE groups displayed reduced proliferative and differentiation capacities. Transcriptomic analysis identified 631 differentially expressed genes between N-P and N-F UCMSCs, with significant enrichment of pathways involved in cell proliferation, differentiation, and EV biology. Network analysis identified CTNNB1 and SRC as key hub genes, and RT-qPCR confirmed differential expression of representative hub genes. In the neonatal GMH model, both N-F- and N-P-derived UCMSCs significantly improved behavioral outcomes and reduced brain injury compared with untreated GMH mice. Although N-P-derived UCMSCs showed a trend toward greater therapeutic benefit, no statistically significant difference was observed between the two treatment groups.

**Conclusion:**

Healthy preterm UCMSCs display distinct biological and paracrine-related properties while retaining neuroprotective effects comparable to that of full-term UCMSCs. In contrast, adverse intrauterine conditions, including gestational diabetes and severe preeclampsia, are associated with impaired UCMSC biological properties. These findings provide new insights into the developmental regulation of UCMSC function and support further investigation of preterm UCMSCs as a promising autologous cell source for regenerative therapies in premature infants.

**Supplementary Information:**

The online version contains supplementary material available at https://doi.org/10.1186/s13287-026-05242-8.

## Background

Mesenchymal stem cells (MSCs) are multipotent progenitor cells that can be derived from sources such as bone marrow, umbilical cord tissue, adipose tissue, lung tissue, dental pulp, and placenta [1]. Among these sources, human umbilical cord-derived mesenchymal stem cells (UCMSCs) have attracted considerable attention because of their high proliferative capacity, low immunogenicity, ready availability, and ethical acceptability [2–4].

In recent years, UCMSCs have been increasingly investigated for their therapeutic effects in neonatal brain injury and other inflammatory or degenerative conditions [5]. Their efficacy is thought to be mediated largely through paracrine mechanisms, particularly the release of extracellular vesicles (EVs), which can modulate immune responses [6], reduce apoptosis [7], and promote tissue regeneration [8]. UCMSC-derived EVs have demonstrated anti-inflammatory and neuroprotective effects in models of hypoxic-ischemic brain injury [9], spinal cord injury [10], osteoarthritis [11], diabetes [12], and lupus nephritis [13]. Notably, intranasal administration has emerged as a promising noninvasive route for UCMSC- or UCMSC-EV-based therapy, especially in neonatal applications [14].

Despite promising preclinical data, most UCMSC and EV studies—as well as current clinical trials—have used cells derived from full-term infants [15]. However, accumulating evidence suggests that MSC properties may vary with developmental age [16]. Stem cell functions, including proliferation, differentiation, and cytokine secretion, are age dependent and may decline with maturation [17]. Preterm UCMSCs may retain developmental-stage-associated characteristics. For example, preterm-derived perinatal MSCs, including umbilical cord blood-derived MSCs, have been shown to provide greater protection against white matter injury in animal models through immunomodulatory mechanisms, suggesting that developmental age may influence therapeutic potential [18]. Nevertheless, the mechanisms and transcriptomic features underlying these differences remain poorly understood.

In particular, although previous studies have suggested developmental age-related differences in the secretome of preterm- and term-derived MSCs, integrated comparisons of UCMSC cellular phenotypes, EV characteristics, transcriptomic signatures, and in vivo neuroprotective effects remain limited. Understanding these differences could have important implications for autologous or allogeneic cell therapy strategies in neonatal populations, especially those at high risk of brain injury.

In this study, we investigated the biological, functional, and transcriptomic differences between UCMSCs derived from preterm and full-term infants. The primary comparison was between uncomplicated preterm infants (N-P) and full-term infants (N-F). In addition, we included two exploratory preterm subgroups—preterm infants with gestational diabetes exposure (P-GD) and preterm infants with severe preeclampsia exposure (P-SE)—to assess whether common maternal complications associated with preterm birth may influence UCMSC characteristics beyond gestational age alone. We evaluated UCMSC proliferative capacity, differentiation potential, EV secretion profiles, and neuroprotective function, and performed RNA sequencing to identify differentially expressed genes and hub regulatory pathways. Our findings provide new insights into gestational age-associated differences in UCMSC biology and their potential relevance for regenerative therapies in preterm infants.

## Methods

### Isolation and expansion of UCMSCs

The Ethics Committee of the Third Affiliated Hospital of Zhengzhou University (2021-158-01) approved this study. Umbilical cord tissue specimens were obtained after written informed consent from mothers undergoing cesarean delivery. All procedures complied with the Declaration of Helsinki and institutional guidelines. Twenty umbilical cord samples were collected from neonates delivered at the Third Affiliated Hospital of Zhengzhou University. The samples were categorized into four groups: (1) N-F, uncomplicated full-term infants (*n* = 5; gestational age ≥ 37 weeks); (2) N-P, uncomplicated preterm infants (*n* = 5; gestational age < 32 weeks); (3) P-GD, preterm infants with gestational diabetes exposure (*n* = 5; gestational age < 32 weeks); and (4) P-SE, preterm infants with severe preeclampsia exposure (*n* = 5; gestational age < 32 weeks). All samples were confirmed to be free of intrauterine infection and congenital malformations (Supplementary Table S1) [19].

Umbilical cord tissue was processed within 24 h of delivery. After washing with phosphate-buffered saline (PBS), Wharton’s jelly was dissected into 1–3 mm³ fragments and cultured in Amniotic Mesenchymal Stem Cell Medium (NC0103, Yocon, China) in 10-cm culture dishes. Cultures were maintained at 37 °C in 5% CO₂. The medium was replaced every two days. When cells reached 80–90% confluence, they were passaged using 0.25% trypsin-EDTA (Gibco, USA). UCMSCs from passages 3 to 5 were used for all experiments unless otherwise stated.

### Flow cytometry for phenotypic characterization

Passage 5 (P5) UCMSCs were harvested, washed twice with PBS, and adjusted to 3 × 10⁶ cells/mL. Aliquots of 100 µL cell suspension (3 × 10⁵ cells per tube) were transferred to 1.5-mL Eppendorf tubes and incubated with antibodies against CD34, CD45, CD73, CD90, and CD105. After incubation, the cells were washed twice with PBS and analyzed using a BD FACSCanto II flow cytometer. At least 20,000 events were acquired for each sample. Unstained cells were used as the negative control.

### Trilineage differentiation assays

Adipogenic differentiation: UCMSCs (P3) were seeded in 6-well plates and induced using MesenCult™ Adipogenic Differentiation Medium (Cat #05412, STEMCELL Technologies, Canada) for 14 days. The medium was changed according to the manufacturer’s instructions. Lipid accumulation was visualized by Oil Red O staining.

Osteogenic differentiation: Cells were cultured in Osteogenic Differentiation Medium (Cat #05465, STEMCELL Technologies) for 21 days. The medium was refreshed according to the manufacturer’s instructions. Calcium deposits were stained with Alizarin Red.

Chondrogenic differentiation: For pellet culture, 2 × 10⁶ UCMSCs (P3) were resuspended in MesenCult™ ACF Chondrogenic Medium (Cat #05445, STEMCELL Technologies) and incubated for 21 days. Differentiated pellets were fixed, embedded in paraffin, sectioned, and stained with Alcian Blue/Nuclear Fast Red.

### Cell proliferation and viability assays

CCK-8 assay: UCMSCs (3 × 10⁴ cells/well) were seeded in 96-well plates and cultured for 7 days. Cell proliferation/viability was measured daily using the Cell Counting Kit-8 assay (Cat. #CA1210-100, Solarbio, China) according to the manufacturer’s instructions, and absorbance was recorded at 450 nm.

EdU incorporation assay: Cell proliferation was further evaluated using the EdU Cell Proliferation Kit (Cat. #C10310-1, RiboBio, China). After a 2-h EdU pulse, cells were fixed and stained according to the manufacturer’s protocol. EdU-positive cells were visualized by fluorescence microscopy and quantified as the percentage of total nuclei.

Apoptosis assay: Apoptosis was assessed using Annexin V-FITC/PI staining with an apoptosis detection kit (BD Pharmingen, USA). Briefly, 1 × 10⁶ cells were incubated with 5 µL Annexin V-FITC and 10 µL propidium iodide in binding buffer for 15 min in the dark, followed by flow cytometric analysis.

### Isolation and characterization of small extracellular vesicles (sEVs)

UCMSCs were cultured to approximately 70–80% confluence and then maintained in serum-free Amniotic Mesenchymal Stem Cell Medium for 48 h. The conditioned medium was collected, and sEVs were isolated by differential ultracentrifugation. Briefly, the conditioned medium was sequentially centrifuged at 300 × g for 10 min, 2,000 × g for 10 min, and 10,000 × g for 30 min to remove cells, debris, and larger vesicles. The resulting supernatant was then ultracentrifuged at 100,000 × g for 70 min at 4 °C. The sEV pellet was resuspended in 50 µL of PBS and stored at − 80 °C until further analysis.

The morphology of sEVs was examined by transmission electron microscopy (TEM). Briefly, 10 µl of fresh isolated sEV suspension was prepared for TEM imaging, and images were obtained using a Hitachi HT7800 TEM system operated at 120 kV.

Particle size distribution and concentration were determined by nanoparticle tracking analysis (NTA) using ZetaView^®^ nanoparticle tracking analysis system (Particle Metrix GmbH, Germany). Samples were diluted 1:1000 in PBS before analysis.

The expression of positive sEV markers (CD9, CD63, TSG101) and negative marker (Calnexin) were examined by western blotting. Briefly, sEVs were lysed with EV-specific lysis buffer (Umibio, #UR33101, Shanghai, China) as described previously [20]. Protein concentration was determined using a bicinchoninic acid (BCA) protein assay, and equal amounts (10 µg) of protein were subjected to western blot analysis.

Surface marker expression was further characterized using the ExoView platform. EV surface marker profiling was performed using ExoView R100 chips (NanoView Biosciences, USA) coated with anti-human CD9, CD63, and CD81 antibodies. After overnight incubation (16 h), the chips were washed, stained with fluorescent antibodies according to the manufacturer’s instructions, and imaged using the ExoView scanner. Data were processed using NanoViewer 2.8.10 software.

### Transcriptome sequencing and bioinformatics analysis

Total RNA was extracted from UCMSCs from the N-F and N-P groups using TRIzol (Invitrogen, USA). All samples were processed using an identical experimental workflow by the same sequencing provider to minimize technique variation. Potential batch effects were assessed by principal component analysis (PCA). RNA integrity was assessed using the Agilent Bioanalyzer 2100, and only samples with an RNA integrity number (RIN) ≥ 7.0 were used for library preparation. Sequencing libraries were prepared and sequenced on the Illumina NovaSeq 6000 platform (LC-Bio Technologies, China), with an average depth of approximately 20 million paired-end reads per sample. The number of biological replicates analyzed in each group was *n* = 5. Raw reads were processed and aligned to the human reference genome GRCh38, and gene expression was quantified for downstream analysis. Differentially expressed gene (DEG) analysis was conducted using DESeq2 software (http://www.bioconductor.org/packages/release/bioc/html/DESeq2.html). Genes with an adjusted *p < 0.05 and |log2FC| ≥ 1 were defined as DEGs. To identify category-specific DEGs, the full DEG list was mapped to predefined GO annotations and literature-supported functional categories related to cell proliferation, differentiation, and exosome biology. These category-specific gene sets were then subjected to Gene Ontology (GO) (http://www.geneontology.org/) and KEGG (http://www.kegg.jp) pathway enrichment analyses.

A protein-protein interaction (PPI) network of DEGs was then established using the STRING database (https://string-db.org/) [21]. Network visualization was performed using Cytoscape software (version 3.7.2; www.cytoscape.org). Hub genes were identified using the CytoHubba plugin in Cytoscape [22], and genes were ranked by maximal clique centrality (MCC) score; the top 10 genes were defined as hub genes.

### Reverse transcription quantitative PCR

A total of 5 × 10^5^ UCMSCs per sample were manually homogenized in RNase-free PBS, and total RNA was extracted using an miRNeasy Mini kit (Cat. RN6801, Aidlab, China) according to the manufacturer’s instructions. RNA concentration and purity were assessed using a spectrophotometer (NanoDrop 2000, Thermo scientific, USA) at 260/280 nm absorbance (*n* = 5/group). For each sample, 1 µg RNA was reverse-transcribed into complementary DNA (cDNA) using a QuantiTect Reverse Transcription kit (Yeasen, China). The reverse transcription was performed as follows: 25 °C for 5 min, 15 min at 55 °C, and 85 °C for 5 min.

Quantitative PCR was performed using QuantiFast SYBR Green PCR Master Mix (Yeasen, China) with primers targeting CTNNB1, SRC, WNT5A, SIX2, ANXA2 and YAP1. The amplification protocol consisted of 45 cycles of 95 °C for 10 s and 60 °C for 30 s. The amount of single-stranded DNA in each cDNA sample was quantified using a Quant-iT OliGreen ssDNA Assay kit (Thermo Fisher Scientific, USA), with fluorescence measured at an excitation wavelength of 480 nm and an emission wavelength of 520 nm. Relative gene expression was normalized according to the cDNA concentration ratio between samples.

### Animals

C57BL/6J mice of both sexes were used in the experiments. Animals were purchased from Beijing Vitroniha Laboratory Animal Technology Co., Ltd (Beijing, China), and bred at the Third Affiliated Hospital of Zhengzhou University, with food and water available ad libitum. All animal experiments were approved by the Animal Care and Ethics Committees of the Third Affiliated Hospital of Zhengzhou University and were conducted in accordance with FELASA guidelines and the 3Rs principles. Animal data were reported in accordance with the ARRIVE 2.0 guidelines [23]. Mouse pups of both sexes within the weight range of 2.7–3.1 g at PND 5 were randomly assigned to either control or GMH groups. Experimenters were blinded to the group assignments. A total of 12 pups were excluded, and 96 pups were included in all experiments. Pups were randomly assigned to each group with stratification by sex. Sample size was determined based on our experimental design and on numbers reported in previous studies [24]. Behavioral assessments and MRI data analysis were performed independently by investigators blinded to group allocation. At the experimental endpoint after MRI, mice were deeply anesthetized with phenobarbital (50 mg/mL). After confirming the absence of the pedal withdrawal reflex, transcardial perfusion was performed through the ascending aorta with phosphate-buffered saline, followed by 4% buffered formaldehyde for 5 min. The brains were then rapidly dissected, post-fixed in 4% buffered formaldehyde at 4 °C for 24 h, dehydrated through graded ethanol and xylene, and embedded in paraffin for histological analysis.

### Germinal matrix hemorrhage induction

At P5, mouse pups were randomly allocated to the Sham and GMH groups. Following anesthesia with isoflurane (3.5% for induction and 2.5% for maintenance) in a mixture of oxygen and nitrogen, pups were injected into the right medial striatum with either collagenase VII (0.15 U in 1 µL saline, Merck KGaA, Darmstadt, Germany) to induce GMH or an equal volume of saline for the Sham group. Injections were administered at 1 µL/min for 2 min at the following stereotaxic coordinates: 2 mm rostral to bregma, 1.5 mm lateral to the midline, and 2 mm in depth, using a 28-gauge needle connected to a 25 µL Hamilton syringe and a CMA/100 microinjection pump, as previously described [25, 26]. After the procedure, pups were allowed to recover on a heating pad at 37 °C and were then returned to their dams until sacrifice at the designated experimental time points.

### UCMSC administration

UCMSCs intended for intranasal delivery were derived from the N-P and N-F groups. Seventy-two GMH pups were randomized into three groups (*n* = 24/group), stratified by sex, and received N-P-derived UCMSCs, N-F-derived UCMSCs, or PBS via the intranasal route. At 14 days post-GMH induction, animals were pretreated intranasally with hyaluronidase (100 U in 6 µL total; 2 droplets of 1.5 µL per nostril; MCE). Thirty minutes later, mice received 5 × 10⁵ UCMSCs suspended in PBS, or PBS alone as vehicle control, by intranasal administration of 2 droplets of 1.5 µL per nostril (6 µL total), as previously described [27].

### Magnetic resonance imaging

Magnetic resonance imaging (MRI) was performed on day 28 post-GMH induction using a 4.7-T preclinical scanner (MR Solutions, Guildford, UK). Coronal T2-weighted images (T2WI) were acquired with the following parameters: repetition time = 5000 ms, echo time = 51 ms, flip angle = 180°, field of view = 25 × 25 mm, matrix = 256 × 256, number of slices = 22, and slice thickness = 0.5 mm. Images were processed using ITK-SNAP 3.8.0 and SimpleITK. Binary masks were generated for the whole brain, ipsilateral hemisphere, and lesion area, and volumes were calculated from voxel spacing in the image metadata. Intact total brain and ipsilateral hemisphere volumes were determined by subtracting lesion volume from the corresponding gross anatomical volumes.

### Behavioral tests

A comprehensive battery of neurobehavioral tests was conducted on days 23 and 25 post-GMH induction to evaluate locomotor activity and motor coordination, as previously described [24]. Prior to testing, animals were acclimatized to the dimly lit testing room for at least 30 min. Equipment was cleaned with 75% ethanol between subjects to eliminate olfactory cues. Data acquisition and analysis were performed using ANY-Maze software (Stoelting, Wood Dale, IL, USA).

Open field test: At 23 days post-GMH, spontaneous locomotor activity was assessed using the open field test (OFT) as previously described. Mice were placed in a white acrylic open-top box (45 × 45 × 45 cm; Global Biotech, Shanghai, China) equipped with an overhead infrared CCD camera and infrared illumination. During the 30-min session, the arena was virtually partitioned into a central zone (22.5 × 22.5 cm) and a peripheral zone. Key parameters, including distance traveled in the center, time spent in the center, number of entries into the central area, and total distance traveled, were quantified.

Rotarod test: Motor coordination and balance were evaluated at 25 days post-GMH using a rotarod apparatus (Global Biotech). Mice were placed on a rotating drum and subjected to an accelerating protocol. Animals were initially habituated at 4 rpm for 5 min, followed by acceleration from 4 to 40 rpm over 1 min. Latency to fall was recorded across three consecutive trials, with a 30-min intertrial interval in the home cage to minimize fatigue.

### Statistical analysis

Data are presented as mean ± standard deviation (SD). Statistical analyses were performed using GraphPad Prism 8 (GraphPad Software, La Jolla, CA, USA). For in vitro experiments, the number of biological replicates is indicated in the corresponding figure legends or method descriptions. Each biological replicate represents an independent umbilical cord donor. Data normality was assessed using the Shapiro-Wilk test prior to statistical analysis. For comparisons among multiple groups at a single time point, one-way analysis of variance (ANOVA) followed by Tukey’s post hoc test was used for normally distributed data, whereas the Kruskal–Wallis test followed by Dunn’s multiple-comparison test was used for non-normally distributed data. For time-course data, such as the CCK-8 proliferation assay, two-way ANOVA was performed where appropriate. For transcriptomic analyses, P values were adjusted using the Benjamini–Hochberg procedure to control the false discovery rate (FDR). All statistical tests were two-sided, and a P value < 0.05 was considered statistically significant.

## Results

### UCMSCs display typical morphology and mesenchymal surface markers

**Fig. 1:**
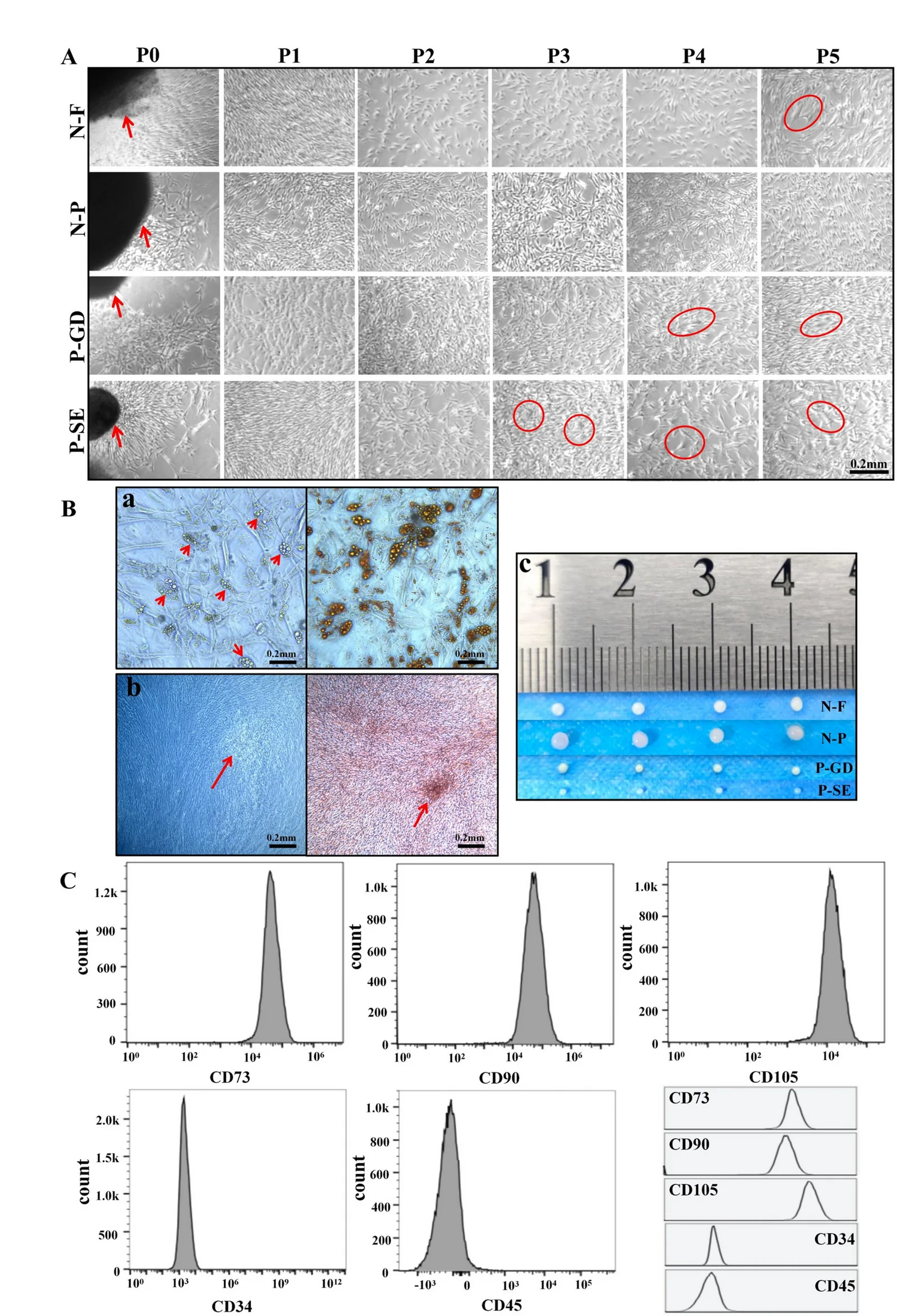
Morphology, trilineage differentiation, and phenotypic characterization of UCMSCs. **A** Morphological changes in UCMSCs from the different groups during passaging. Red arrows indicate umbilical cord tissue explants, and red circles indicate differentiated cells. **B** Trilineage differentiation assays. **a** Adipogenesis: red arrows indicate lipid droplets, and positive Oil Red O staining appears as orange lipid droplets. **b** Osteogenesis: arrows indicate calcium deposits secreted by osteoblasts, and positive Alizarin Red staining marks calcium deposition. **c** Chondrogenesis: representative differentiated cartilage pellets. **C** Flow cytometry analysis showing high expression of mesenchymal markers (CD73, CD90, and CD105; >98% positive) and low expression of hematopoietic markers (CD34 and CD45; <1% positive)

All UCMSCs isolated from the four groups (N-F, N-P, P-GD, and P-SE) adhered to the plastic surface and exhibited fibroblast-like spindle morphology under light microscopy. Notably, spontaneous morphological differentiation appeared earlier in full-term UCMSCs (P5) than in the preterm groups. Differentiated cells appeared even earlier in the P-GD (P4) and P-SE (P3) groups (Fig. 1A). Trilineage differentiation assays confirmed adipogenic, osteogenic, and chondrogenic potential in all groups. The N-P group demonstrated distinct chondrogenic differentiation, with more robust cartilage pellet formation and stronger Alcian Blue staining (Fig. 1B). Flow cytometric analysis confirmed high expression (> 98%) of MSC markers CD73, CD90, and CD105 and low expression (< 1%) of hematopoietic markers CD34 and CD45 across all samples (Fig. 1C).

### Preterm UCMSCs Exhibit a Higher CCK-8 Growth/Viability Signal

**Fig. 2:**
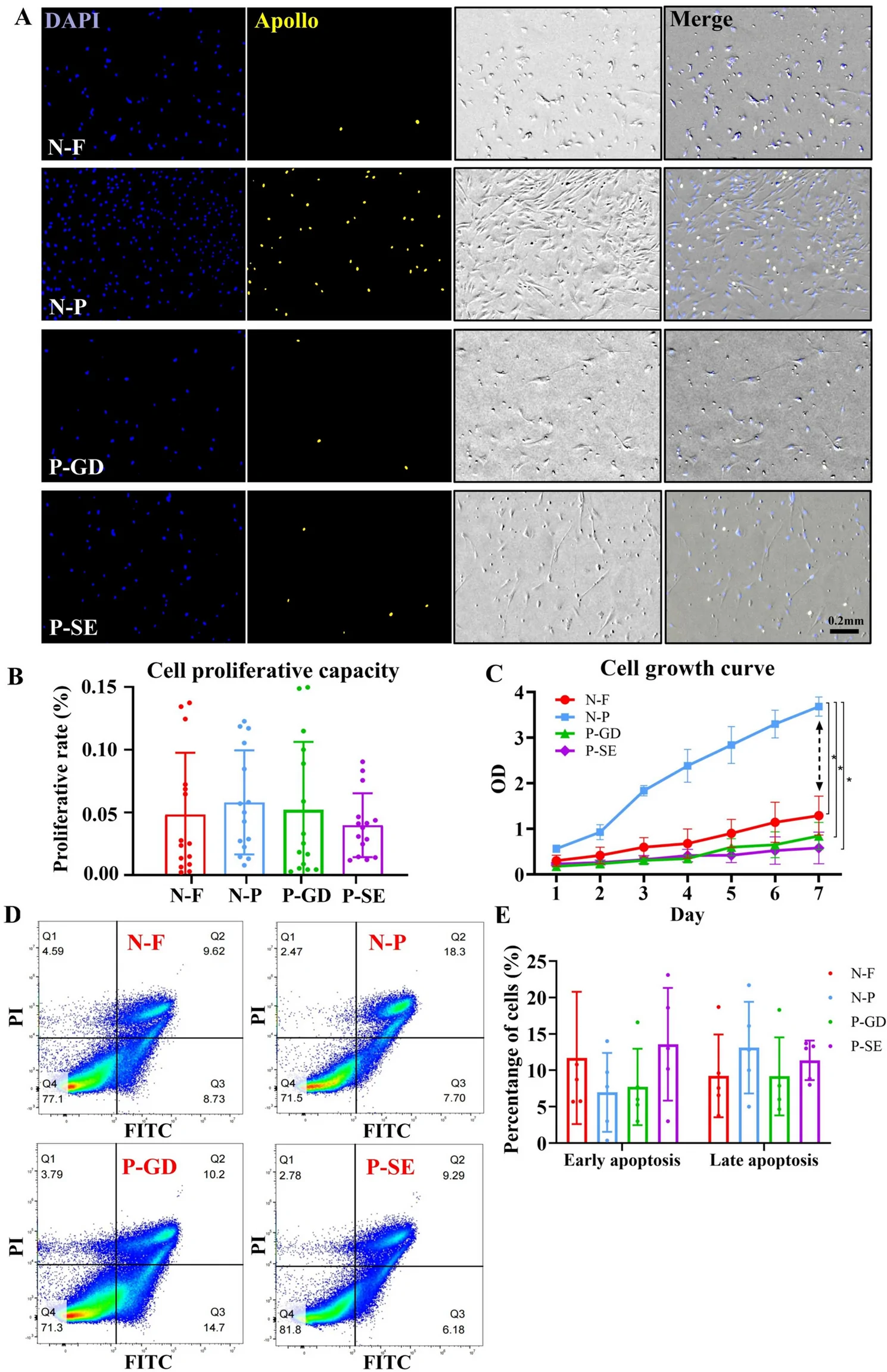
Proliferation and apoptosis of UCMSCs. **A**-**B** EdU incorporation assay (*n* = 5/group, 5 biological donors × 3 technical replicates, mean ± SD, N-F vs. N-P, *p* = 0.568; N-P vs. P-GD, *p* = 0.737; N-P vs. P-SE, *p* = 0.158). **C** CCK-8 proliferation assay (*n* = 5/group, 5 biological donors × 3 technical replicates, mean ± SD, *p < 0.05). Data were analyzed by two-way ANOVA followed by Tukey’s post hoc test. **D**-**E** Annexin V/PI apoptosis analysis (*n* = 5/group, early apoptosis: N-F vs. N-P, *p* = 0.347; N-P vs. P-GD, *p* = 0.846; N-P vs. P-SE, *p* = 0.162. Late apoptosis: N-F vs. N-P, *p* = 0.338; N-P vs. P-GD, *p* = 0.314; N-P vs. P-SE, *p* = 0.56). Data were analyzed by one-way ANOVA with Tukey’s post hoc test

Cell proliferation was first assessed using EdU incorporation. Compared with the other three groups, the N-P group showed a numerically higher proliferative capacity, although the difference was not statistically significant (N-F vs. N-P, *p* = 0.568; N-P vs. P-GD, *p* = 0.737; N-P vs. P-SE, *p* = 0.158) (N-F: 0.048 ± 0.049, N-P: 0.058 ± 0.041, P-GD: 0.052 ± 0.054, P-SE: 0.04 ± 0.025 (Fig. 2A-B). The CCK-8 assay showed a higher growth/viability signal in the N-P group over 7 days, with peak absorbance on day 7 (OD450 = 3.68 ± 0.21) compared with that in the N-F group (1.29 ± 0.43, *p* < 0.05), in the P-GD group (0.84 ± 0.3, *p* < 0.05) and in the P-SE group (0.58 ± 0.35, *p* < 0.05) (Fig. 2C). Apoptosis rates, as measured by Annexin V/PI flow cytometry, did not differ significantly across groups (Early apoptosis: N-F vs. N-P, *p* = 0.347; N-P vs. P-GD, *p* = 0.846; N-P vs. P-SE, *p* = 0.162. Late apoptosis: N-F vs. N-P, *p* = 0.338; N-P vs. P-GD, *p* = 0.314; N-P vs. P-SE, *p* = 0.56), indicating that the enhanced cell viability observed in the N-P group was not attributable to reduced apoptosis (Fig. 2D-E).

### Preterm UCMSCs Secrete More Abundant Extracellular Vesicles

**Fig. 3:**
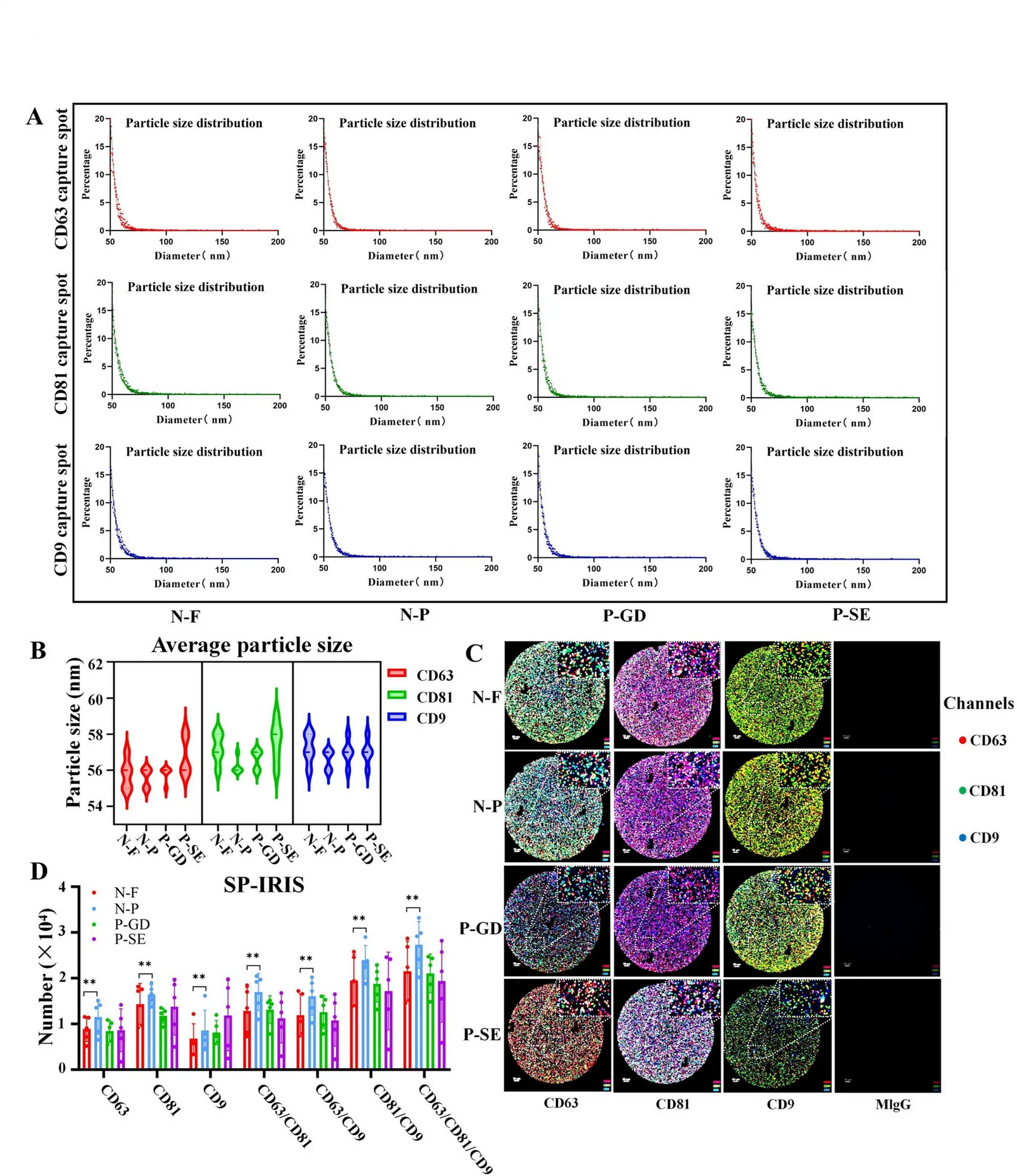
Characterization of EVs derived from UCMSCs. **A** Representative nanoparticle tracking analysis (NTA) showing the size distribution of EVs isolated from UCMSCs. **B** Quantification of the mean particle diameter of CD9⁺, CD63⁺, and CD81⁺ EVs. No significant differences in EV size were observed among groups. **C** Quantification of CD9⁺, CD63⁺, and CD81⁺ EV abundance measured by ExoView analysis. N-P-derived UCMSCs secreted significantly higher numbers of CD9⁺, CD63⁺, and CD81⁺ EVs than N-F-derived UCMSCs. P values for comparisons among EV surface markers were adjusted using the Benjamini–Hochberg false discovery rate (FDR) procedure. **D** Representative ExoView fluorescence images demonstrating the increased abundance of CD9⁺, CD63⁺, and CD81⁺ EVs in the N-P group. Data are presented as mean ± SD (*n* = 5 biological replicates per group). Statistical analyses were performed using one-way ANOVA followed by Tukey’s multiple-comparison test. Adjusted P values after FDR correction are indicated in the figure. *P < 0.05, **P < 0.01

EVs were successfully isolated from all UCMSC groups and analyzed by SP-IRIS and NTA. EV characterization is shown in Supplementary Fig. S1 and Fig. 3A. Although there were no statistically significant differences in mean particle size among groups (CD63⁺ EVs: 55.6–56.8 nm, (N-F: 55.8 ± 10, N-P: 55.6 ± 8.4, P-GD: 55.8 ± 11, P-SE: 56.8 ± 11.8. N-F vs. N-P, *p* = 0.67; N-P vs. P-GD, *p* = 1; N-P vs. P-SE, *p* = 0.068); CD81⁺ EVs: 56.2–57.6 nm, (N-F: 57.2 ± 11.2, N-P: 56.2 ± 9.4, P-GD: 56.6 ± 12, P-SE: 57.6 ± 13.2. N-F vs. N-P, *p* = 0.06; N-P vs. P-GD, *p* = 0.242; N-P vs. P-SE, *p* = 0.068); CD9⁺ EVs: 56.6–57.2 nm, (N-F: 57.2 ± 10.8, N-P: 56.6 ± 9.8, P-GD: 57 ± 11.8, P-SE: 57 ± 12. N-F vs. N-P, *p* = 0.223; N-P vs. P-GD, *p* = 0.347; N-P vs. P-SE, *p* = 0.347)), the N-P group exhibited the smallest average EV diameter overall (Fig. 3A-B). More strikingly, the N-P group secreted significantly more CD63⁺, CD81⁺, and CD9⁺ EVs than the N-F group (*p* < 0.01) (Fig. 3C-D). Immunocolocalization analysis further confirmed a higher abundance of multivalent EVs (CD63⁺/CD81⁺/CD9⁺) in the N-P group, suggesting enhanced exosomal biogenesis or release.

### Distinct transcriptomic signatures and pathway enrichment in preterm UCMSCs

**Fig. 4:**
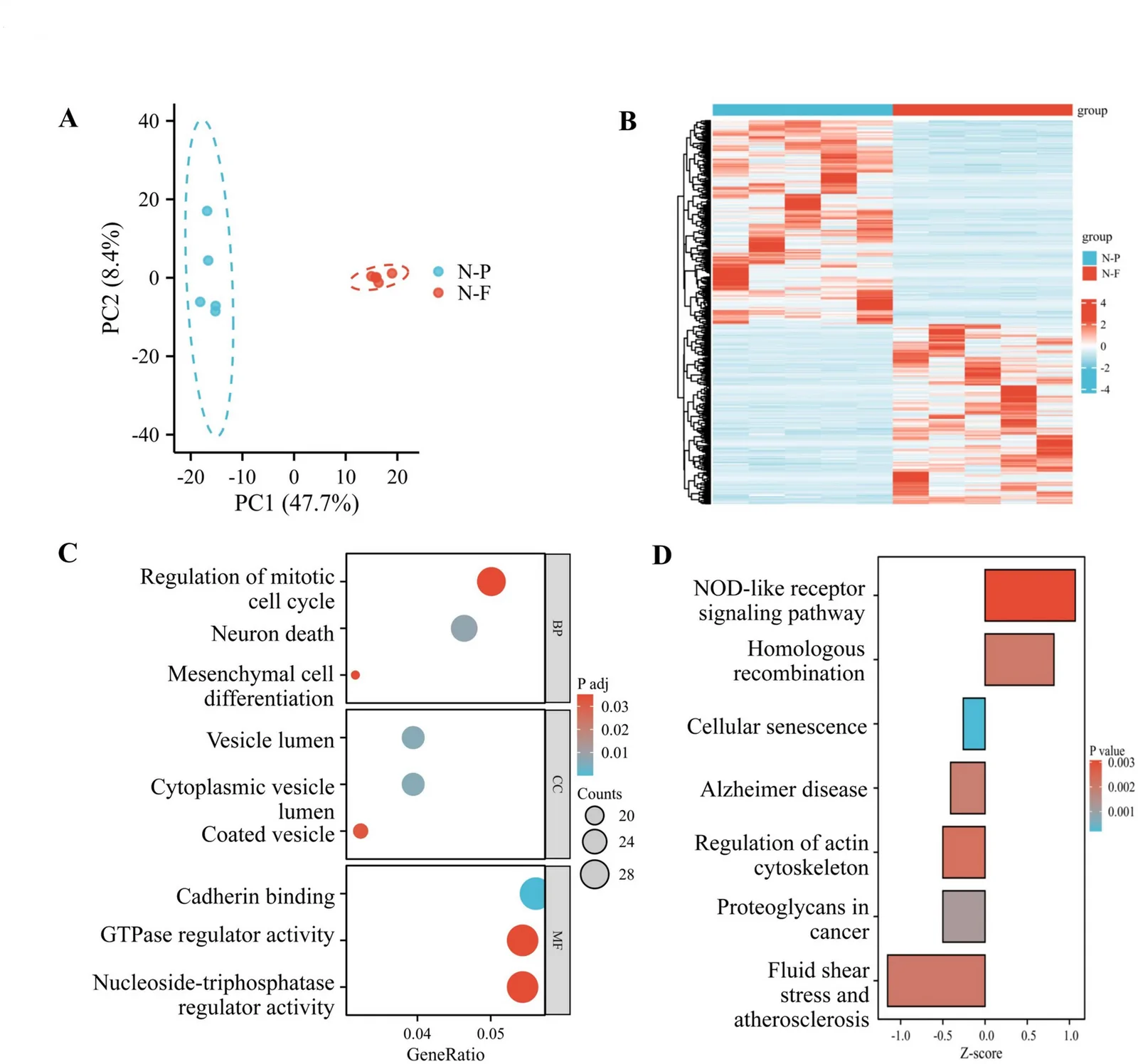
Differential gene expression analysis between N-P and N-F UCMSCs. **A** Principal component analysis revealed that the gene expression profiles of the N-P group (blue) were distinct from those of the N-F group (red). **B** Heatmap showing the expression of 631 DEGs in each group. **C** GO analysis of DEGs. **D** KEGG analysis of DEGs

RNA sequencing comparing N-F and N-P UCMSCs identified 631 differentially expressed genes (DEGs), of which 335 were upregulated and 296 were downregulated in the N-P group (adjusted *p* < 0.05, |log2FC| ≥ 1) (Fig. 4A-B, Supplementary Data S1). Principal component analysis (PCA) confirmed distinct transcriptomic signatures between the groups (PC1 = 47.7%, PC2 = 8.4%). Gene Ontology (GO) enrichment analysis revealed significant enrichment of biological processes related to cell cycle regulation, chondrocyte development, neuron death, and vesicle-mediated transport (Fig. 4C, Supplementary Data S2). In addition, KEGG pathway analysis highlighted the Wnt, TGF-β, Rap1, and Hippo signaling pathways, which are implicated in MSC proliferation and differentiation (Fig. 4D, Supplementary Data S3).

**Fig. 5:**
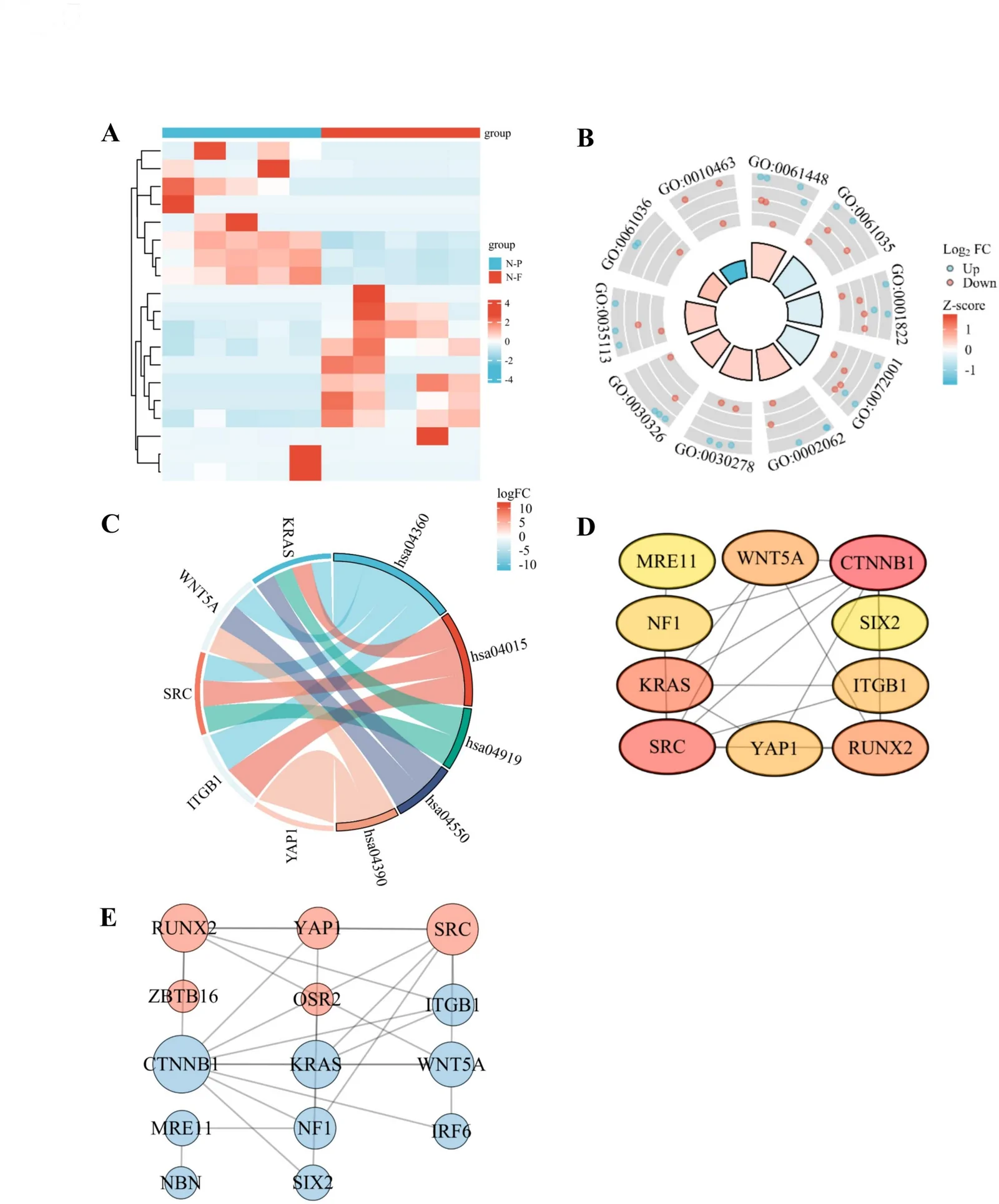
Cell proliferation-related hub genes in N-P UCMSCs. **A** Heatmap of CPRDEGs. **B** GO enrichment analysis of CPRDEGs. **C** KEGG enrichment analysis of CPRDEGs. **D** Top 10 hub genes ranked by MCC in the PPI network. Color indicates rank: CTNNB1, SRC, KRAS, RUNX2, WNT5A, ITGB1, YAP1, NF1, MRE11, and SIX2 (top 1 to 10). **E** Full PPI network of category-specific DEGs. Red indicates upregulation and blue indicates downregulation. Node size indicates the absolute value of |log2FC|, and edge size indicates the co-expression score. CPRDEGs: cell proliferation-related differentially expressed genes

Among the 631 DEGs, 19 were related to cell proliferation (Fig. 5A, Supplementary Data S1), with enriched GO terms such as “positive regulation of cartilage development” and “ossification” (Fig. 5B, Supplementary Data S2). KEGG pathway analysis indicated enrichment of the Rap1 signaling pathway and pathways regulating pluripotency (Fig. 5C, Supplementary Data S3). PPI network analysis identified CTNNB1 and SRC as central hub genes, both of which are implicated in stem cell renewal and proliferation (Fig. 5D-E, Supplementary Data S4).

**Fig. 6:**
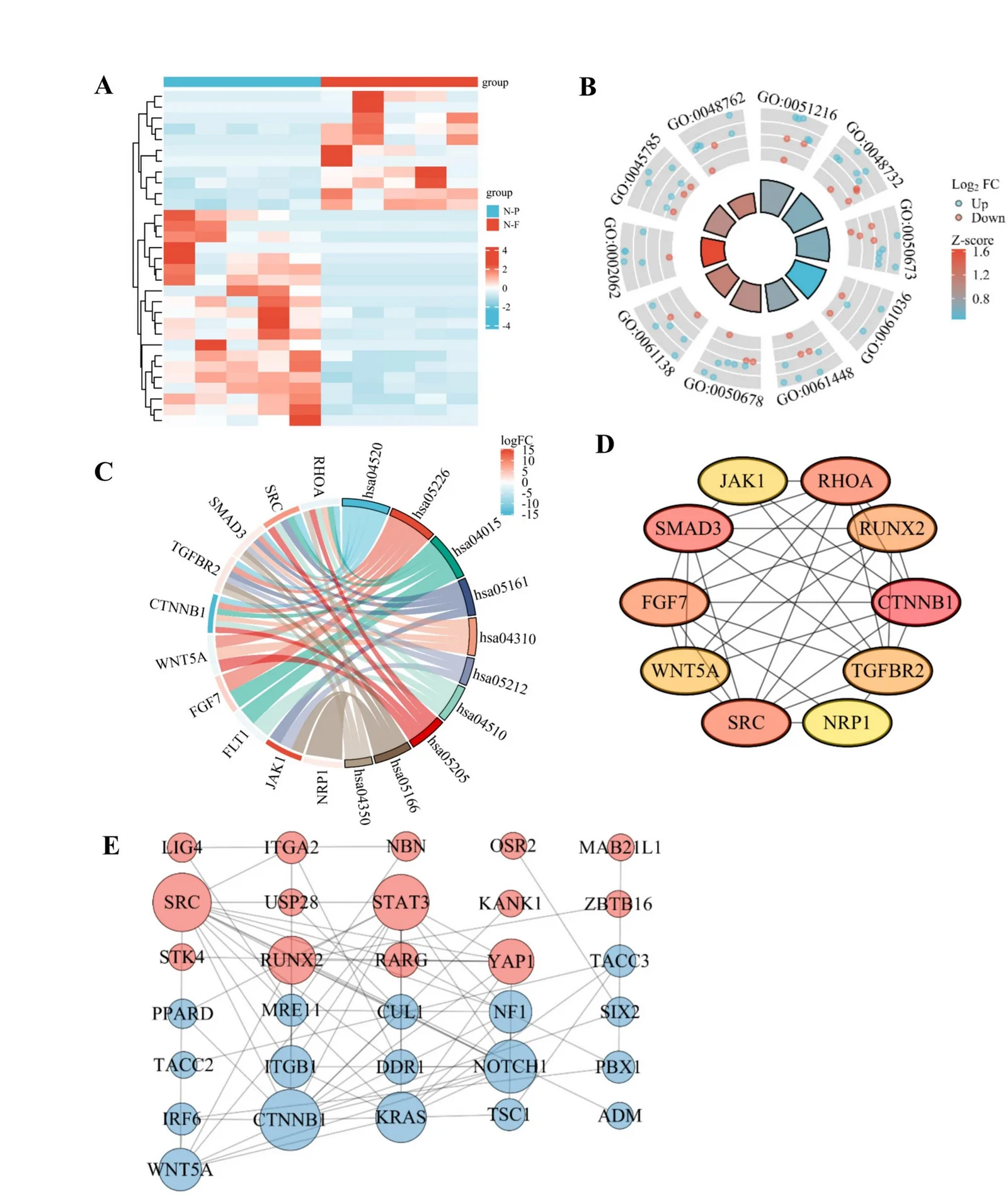
Cell differentiation-related hub genes in N-P UCMSCs. **A** Heatmap of CDRDEGs. **B** GO enrichment analysis of CDRDEGs. **C** KEGG enrichment analysis of CDRDEGs. **D** Top 10 hub genes ranked by MCC in the PPI network. Color indicates rank: CTNNB1, SMAD3, SRC, RHOA, FGF7, RUNX2, TGFBR2, WNT5A, JAK1, and NRP1 (top 1 to 10). **E** Full PPI network of category-specific DEGs. CDRDEGs: cell differentiation-related differentially expressed genes

Additionally, 31 DEGs were linked to differentiation, particularly chondrogenesis (Fig. 6A-C, Supplementary Data S1-3), with SMAD3, WNT5A, and TGFBR2 emerging as key hub genes and supporting the observed differentiation potential of N-P UCMSCs (Fig. 6D-E, Supplementary Data S4).

**Fig. 7:**
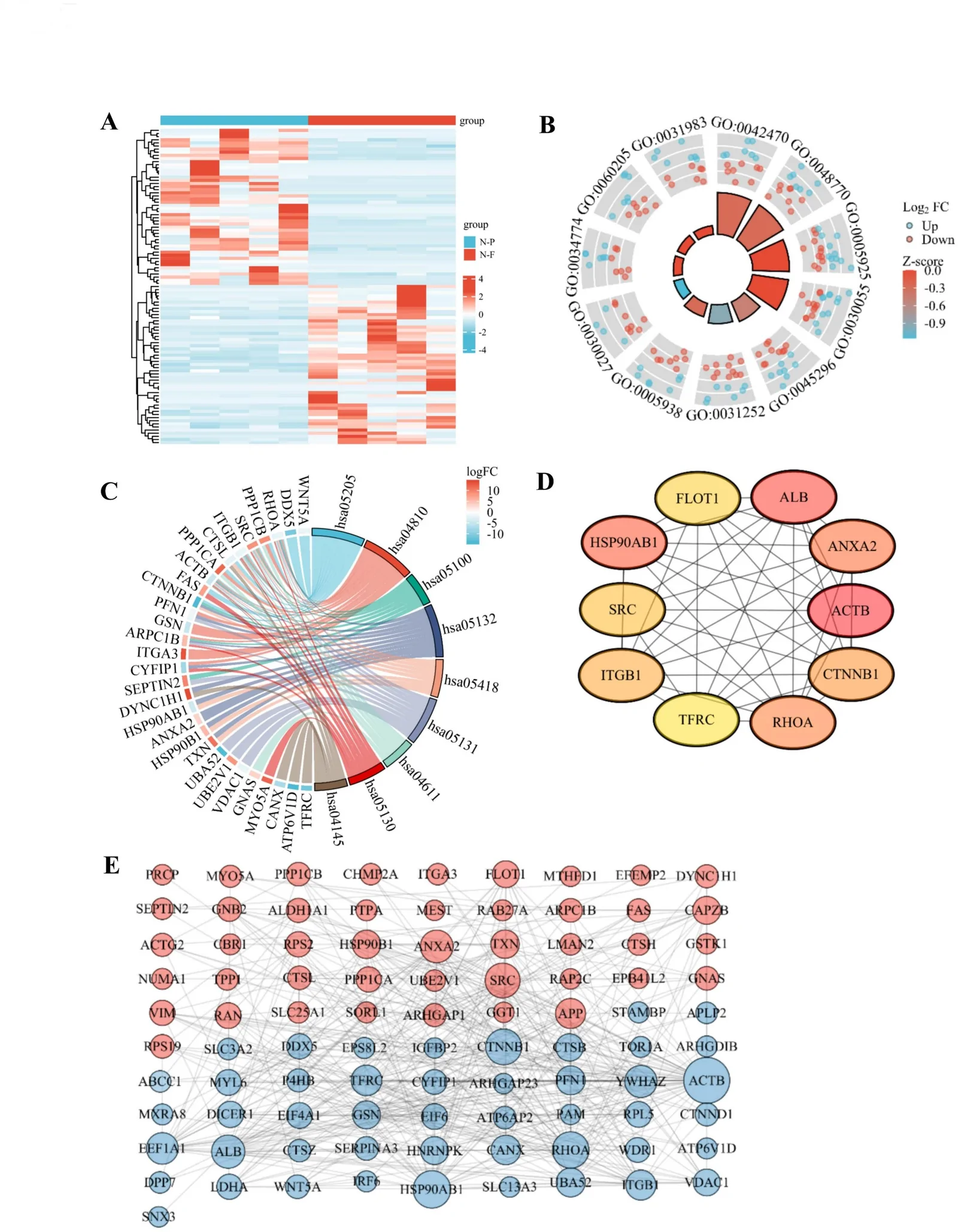
Exosome-related hub genes in N-P UCMSCs. **A** Heatmap of ERDEGs. **B** GO enrichment analysis of ERDEGs. **C** KEGG enrichment analysis of ERDEGs. **D** Top 10 hub genes ranked by MCC in the PPI network. Color indicates rank: ACTB, ALB, HSP90AB1, ANXA2, RHOA, CTNNB1, ITGB1, SRC, FLOT1, and TFRC (top 1 to 10). **E** Full PPI network of category-specific DEGs. ERDEGs: exosome-related differentially expressed genes

A total of 102 exosome-related DEGs were identified (Fig. 7A, Supplementary Data S1). GO analysis revealed enrichment in vesicle lumen- and organelle membrane-related processes, while KEGG analysis pointed to platelet activation and secretion pathways (Fig. 7B-C, Supplementary Data S2-3). PPI analysis identified ACTB, ANXA2, and FLOT1 as central hub genes involved in exosome regulation (Fig. 7D-E, Supplementary Data S4).

To validate the RNA-seq results, RT-qPCR was performed for the two hub genes identified by network analysis (CTNNB1 and SRC) together with four biologically relevant candidate genes (WNT5A, SIX2, ANXA2, and YAP1). The RT-qPCR results showed expression patterns consistent with the RNA-seq data, confirming the differential expression of these genes and supporting the reliability of the transcriptomic analysis (Supplementary Fig. S2).

### Behavioral and MRI assessment of UCMSC treatment after GMH

**Fig. 8:**
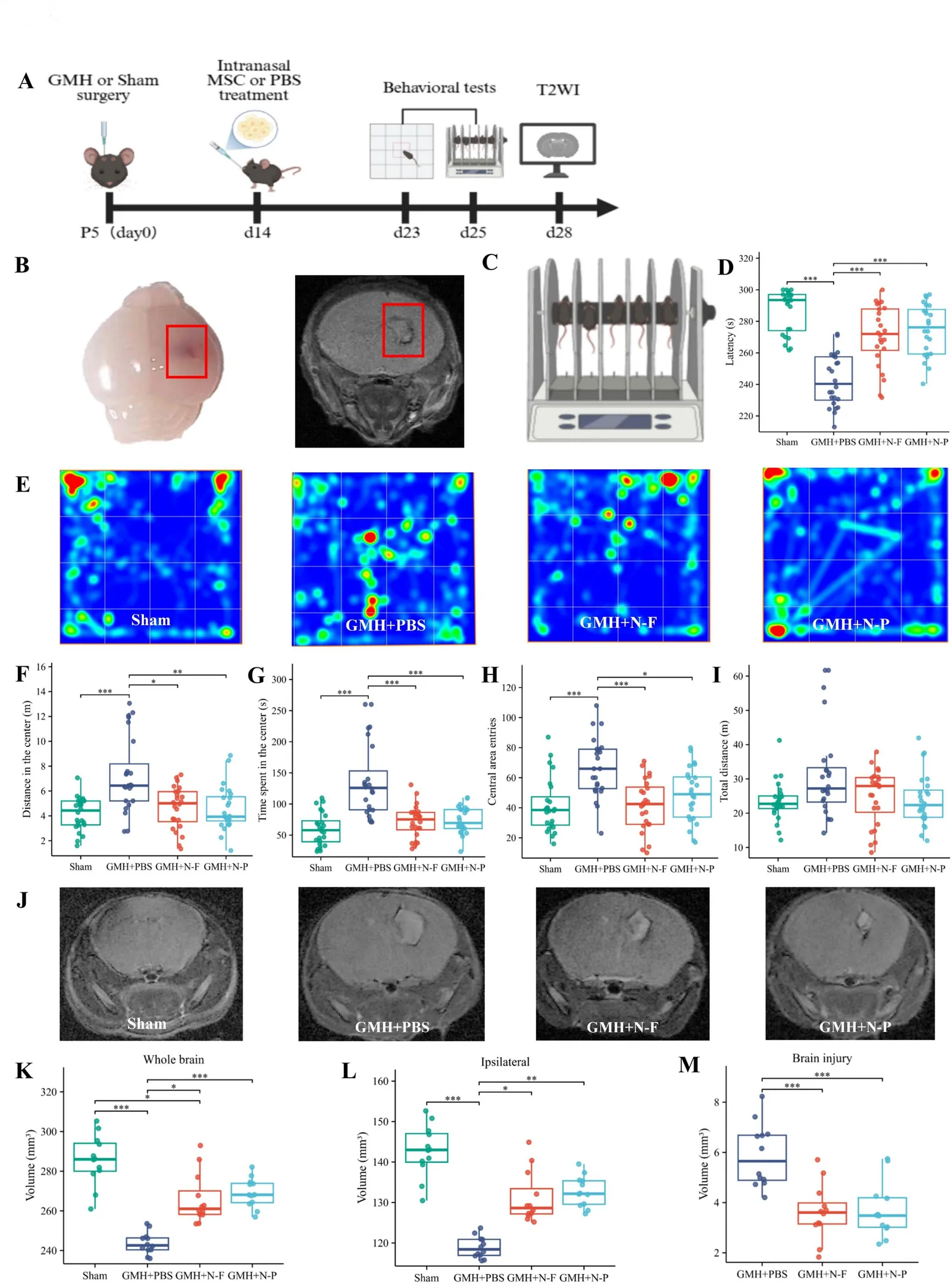
Behavioral and MRI assessment of UCMSC treatment after GMH. **A** Schematic timeline illustrating GMH induction, intranasal administration of UCMSCs or PBS, neurobehavioral testing, and MRI scanning. **B** Representative T2-weighted MRI image and schematic indicating the hematoma location (red box) in the right striatum. **C** Schematic of the rotarod apparatus used to assess motor coordination and balance. **D** Rotarod performance shown as latency to fall among the Sham, GMH + PBS, GMH + N-F, and GMH + N-P groups. **E** Representative open field test (OFT) heat maps showing locomotor trajectories; warmer colors indicate increased time spent in specific zones. **F**-**I** Quantification of OFT parameters, including distance traveled in the center, time spent in the center, number of entries into the central area, and total distance traveled among the Sham, GMH + PBS, GMH + N-F, and GMH + N-P groups (*n* = 24 mice/group). **J** Representative T2-weighted MRI images of brain injury in each group on day 28 post-GMH. **K**-**M** Quantification of whole-brain, ipsilateral hemisphere and brain lesion volume (*n* = 12 mice/group). Both N-F- and N-P-derived UCMSCs significantly improved behavioral and MRI outcomes compared with the GMH + PBS group. Although N-P-derived UCMSCs showed numerical trends toward greater improvement in some behavioral and MRI parameters, no statistically significant differences were detected between the N-P and N-F treatment groups for any behavioral or MRI outcome. Data are presented as mean ± SD. Statistical significance is indicated in the figure. *p < 0.05, **p < 0.01, ***p < 0.001

To evaluate neuroprotective efficacy, a neonatal GMH mouse model was established and treated intranasally with N-P-derived UCMSCs, N-F-derived UCMSCs, or PBS on day 14 after induction (Fig. 8A-B). Behavioral testing showed that both UCMSC-treated groups significantly improved rotarod and OFT performance compared with the GMH group (Fig. 8C-I). Although the N-P group consistently exhibited numerically greater functional improvement than the N-F group, no statistically significant differences were observed between the two treatment groups (all *P* > 0.05).

T2-weighted MRI on day 28 post-GMH demonstrated reduced lesion severity in both treatment groups (Fig. 8J-M). Volumetric analysis further showed increased whole-brain and ipsilateral hemisphere volumes and reduced lesion volume in both UCMSC-treated groups compared with the GMH group. No statistically significant differences were detected between the N-P and N-F groups in whole-brain volume (269.13 ± 7.43 vs. 265.93 ± 12.74 mm³, *P* = 0.99), ipsilateral hemispheric volume (132.72 ± 3.80 vs. 131.15 ± 6.34 mm³, *P* = 1.00), or lesion volume (3.62 ± 1.11 vs. 3.69 ± 1.10 mm³, *P* = 0.99). Nevertheless, N-P-derived UCMSCs consistently showed a trend toward greater preservation of brain volume and reduced lesion size, although these differences did not reach statistical significance.

## Discussion

This study demonstrates that UCMSCs derived from preterm infants, especially those from uncomplicated pregnancies (N-P group), exhibit distinct biological characteristics compared with full-term UCMSCs. These characteristics include higher CCK-8 growth/viability signal, improved chondrogenic differentiation potential, and increased EV secretion. Transcriptomic profiling identified distinct gene expression patterns in preterm UCMSCs, with functional enrichment in pathways associated with stemness, neurodevelopment, and exosome biogenesis. Hub gene analysis further highlighted CTNNB1 and SRC as central regulators of these biological differences.

The enhanced functionality of preterm UCMSCs is consistent with previous studies suggesting that MSC characteristics are age dependent. MSCs from younger sources tend to have greater proliferative capacity, differentiation potential, and anti-inflammatory effects because of developmental immaturity and increased expression of stemness-related markers [17, 28]. Our findings corroborate those of Huang et al. [16], who reported that preterm UCMSCs migrated faster and achieved higher confluence in culture than term UCMSCs. The increased EV output observed in N-P UCMSCs may be relevant for therapeutic application, given the growing evidence that UCMSC-derived EVs contribute to neuroprotection and immunomodulation in neonatal brain injury models [10, 14]. Although we observed a trend toward smaller EV size in N-P UCMSCs, this finding should be interpreted cautiously.

To better understand the biological basis underlying these developmental-stage-associated differences, we performed transcriptomic profiling. RNA sequencing revealed 631 differentially expressed genes (DEGs) between the N-P and N-F groups, including 19 genes related to proliferation, 31 related to differentiation, and 102 related to exosome pathways. Notably, CTNNB1 (β-catenin) and SRC were identified as central hub genes spanning all three functional categories. CTNNB1 serves as a master regulator of the Wnt signaling pathway, which is crucial for MSC proliferation and osteogenic differentiation [29–31], while SRC, a non-receptor tyrosine kinase, plays a vital role in intracellular signaling pathways governing MSC differentiation and extracellular vesicle (EV) release [32]. The prominent enrichment of the Wnt, TGF-β, and Rap1 signaling pathways in our KEGG analysis further supports a robust regulatory network that may contribute to the distinct functional profile of preterm UCMSCs [33–35].

To strengthen these transcriptomic findings, RT-qPCR validation confirmed that CTNNB1, SRC, ANXA2, WNT5A, SIX2, and YAP1 exhibited expression patterns consistent with the RNA-seq data. These genes have previously been implicated in the regulation of stem cell proliferation, differentiation, and extracellular vesicle biology, suggesting that they may contribute to the distinct functional characteristics observed in preterm UCMSCs. However, the present data are correlative and do not establish direct causal relationships. Previous studies have implicated CTNNB1 and SRC in Wnt signaling, stem cell maintenance, and vesicle biology [29–31], whereas ANXA2, WNT5A, SIX2, and YAP1 have been associated with extracellular vesicle regulation, differentiation, and stem cell function [36–43]. Together, these transcriptomic and validation data suggest that developmental stage is associated with coordinated regulation of pathways involved in stem cell maintenance, differentiation, and extracellular vesicle biology.

Previous research has suggested that the neuroprotective mechanism of MSCs in neonatal brain injury may involve stimulation of regenerative processes rather than inhibition of the initial injury cascade [26, 44, 45]. In addition, evidence suggests that intranasal full-term UCMSC therapy is neuroprotective against GMH-induced white matter injury when administered during the repair phase [46]. Our preliminary data demonstrated a precipitous decline in ipsilateral IBA1^+^CD68^+^ activated microglia on day 14 post-GMH, identifying this time point as a potential onset of the repair phase [24]. To determine whether these molecular and cellular differences translate into therapeutic benefit, we next evaluated the neuroprotective efficacy of N-P- and N-F-derived UCMSCs in a neonatal GMH model. In this model, both N-F- and N-P-derived UCMSCs significantly improved open field and rotarod performance and reduced structural brain injury on T2-weighted MRI compared with the GMH group. These findings provide direct in vivo evidence that the biological differences observed between preterm and term UCMSCs are associated with neuroprotective effects rather than relying solely on bioinformatic analyses. Importantly, although N-P UCMSCs showed a consistent numerical trend toward greater behavioral and MRI improvement than N-F UCMSCs, the difference between these two treatment groups did not reach statistical significance. Therefore, the present data demonstrate comparable neuroprotective efficacy of the two UCMSC sources under the conditions tested, while a therapeutic advantage of preterm-derived UCMSCs remains unproven. It is worth noting that the animal experiment was designed and powered primarily to detect therapeutic effects of UCMSC treatment compared with GMH controls, rather than to detect differences between N-P- and N-F-derived UCMSCs. No a priori sample size calculation was performed specifically for the comparison between the N-P and N-F treatment groups. Accordingly, the absence of a statistically significant difference between N-P and N-F should not be interpreted as proof of equivalence, and larger adequately powered studies will be required to determine whether clinically meaningful differences exist between these two UCMSC sources. Although the precise molecular mechanisms remain to be fully elucidated, our findings suggest that UCMSCs alleviate GMH-induced white matter injury and improve long-term neurological function, potentially by promoting oligodendrocyte maturation, enhancing myelination, and attenuating secondary neuroinflammation [24].

UCMSCs from pregnancies complicated by gestational diabetes (P-GD) or severe preeclampsia (P-SE) showed reduced proliferative and differentiation capacities in the exploratory analysis. This may reflect intrauterine stress, chronic hypoxia, or inflammatory priming that negatively affects fetal stem cell development [47]. These findings are consistent with prior transcriptomic studies of UCMSCs under adverse maternal conditions [48] and emphasize the importance of donor screening in clinical UCMSC applications. However, these findings should be interpreted cautiously because of the small sample size and potential confounding by gestational age and maternal disease status. Importantly, the differences observed between the N-P and N-F groups are interpreted primarily as gestational age-associated effects, whereas the altered features of the P-GD and P-SE groups are more appropriately attributed to maternal pregnancy complications rather than gestational age alone.

While the study includes multiple experimental approaches, some limitations should be acknowledged. The sample size, although reasonable for cell-based experiments, limits generalizability and statistical power for subgroup analyses. A broader gestational-age stratification, including extremely preterm, early preterm, moderate preterm, and late preterm infants, would provide a more refined assessment of developmental-stage effects on UCMSC biology; however, the present study was designed primarily to compare very preterm infants (< 32 weeks) with full-term infants while treating pregnancy-complication subgroups as exploratory cohorts. Although the present study included differentiation assays, EV characterization, transcriptomic profiling, and in vivo neuroprotective validation, direct assessment of immunomodulatory function and EV bioactivity in target neural or immune cells was not performed and warrants further investigation. Additionally, RNA-seq was performed only in the N-F and N-P groups; inclusion of P-GD and P-SE samples would further elucidate the effects of maternal complications on gene expression. Technical limitations related to EV isolation and quantification variability should also be acknowledged. Nonetheless, multiple methods (NTA, SP-IRIS, and transcriptomics) converged to support the findings.

There is growing interest in using UCMSCs and their EVs in clinical applications for neonatal diseases, including bronchopulmonary dysplasia and hypoxic-ischemic encephalopathy [48–50]. However, all current clinical trials have used UCMSCs derived from full-term infants. Our results suggest that preterm UCMSCs may represent a promising candidate source for autologous or allogeneic cell therapy in preterm-related brain injury, particularly because they demonstrated higher CCK-8 growth/viability signal, chondrogenic differentiation, EV secretion, and measurable neuroprotective activity in vivo. However, because the difference in neuroprotective efficacy between N-P- and N-F-derived UCMSCs did not reach statistical significance in the present study, larger studies are needed before concluding that preterm UCMSCs are therapeutically distinct from full-term UCMSCs. Notably, EVs derived from Wharton’s jelly MSCs have shown strong neuroprotective and anti-inflammatory effects in rodent models [14, 51], and preclinical data suggest their potential to alleviate perinatal white matter injury [18]. Given the evidence of increased EV secretion in N-P UCMSCs, our findings provide a rationale for future studies of preterm-derived EV-based therapies. However, whether these EV differences directly contribute to neuroprotection remains unknown. Future work should include functional assays of preterm UCMSC-derived EVs in models of neuroinflammation or white matter injury, proteomic and miRNA profiling of EV cargo, and preclinical safety and biodistribution studies.

## Conclusions

Preterm UCMSCs exhibit distinct biological and paracrine-related properties and exert neuroprotective effects comparable to those of full-term UCMSCs in a neonatal GMH model. These findings suggest developmental-stage-associated molecular differences are compatible with preserved therapeutic efficacy, although larger studies are required to determine whether meaningful functional differences exist between preterm- and term-derived UCMSCs.

## Electronic Supplementary Material

Below is the link to the electronic supplementary material.

Supplementary Material 1.

Supplementary Material 2.

Supplementary Material 3.

Supplementary Material 4.

Supplementary Material 5.

Supplementary Material 6.

Supplementary Material 7.

## Data Availability

All data generated or analyzed during this study are included in this published article and its supplementary information files.

## Abbreviations

UCMSCs: Umbilical cord mesenchymal stem cells
MSCs: Mesenchymal stem cells
EVs: Extracellular vesicles
UCMSC-EVs: Umbilical cord mesenchymal stem cell-derived extracellular vesicles
N-F: Uncomplicated full-term
N-P: Uncomplicated preterm
P-GD: Preterm with gestational diabetes
P-SE: Preterm with severe preeclampsia
PBS: Phosphate-buffered saline
GO: Gene Ontology
KEGG: Kyoto Encyclopedia of Genes and Genomes
DEGs: Differentially expressed genes
PPI: Protein-protein interaction
CPRDEGs: Cell proliferation-related differentially expressed genes
CDRDEGs: Cell differentiation-related differentially expressed genes
ERDEGs: Exosome-related differentially expressed genes
