# Supplementary material for: Gestational age–associated differences in human umbilical cord mesenchymal stem cells: biological, paracrine, and neuroprotective properties
Source: Stem Cell Res Ther. 2026 Aug 12;17:414. doi: 10.1186/s13287-026-05242-8 (PMC13746429; doi:10.1186/s13287-026-05242-8)
Supplement: Supplementary file 1 [file 13287_2026_5242_MOESM1_ESM.pdf]

Supplementary material for Fig. S1B

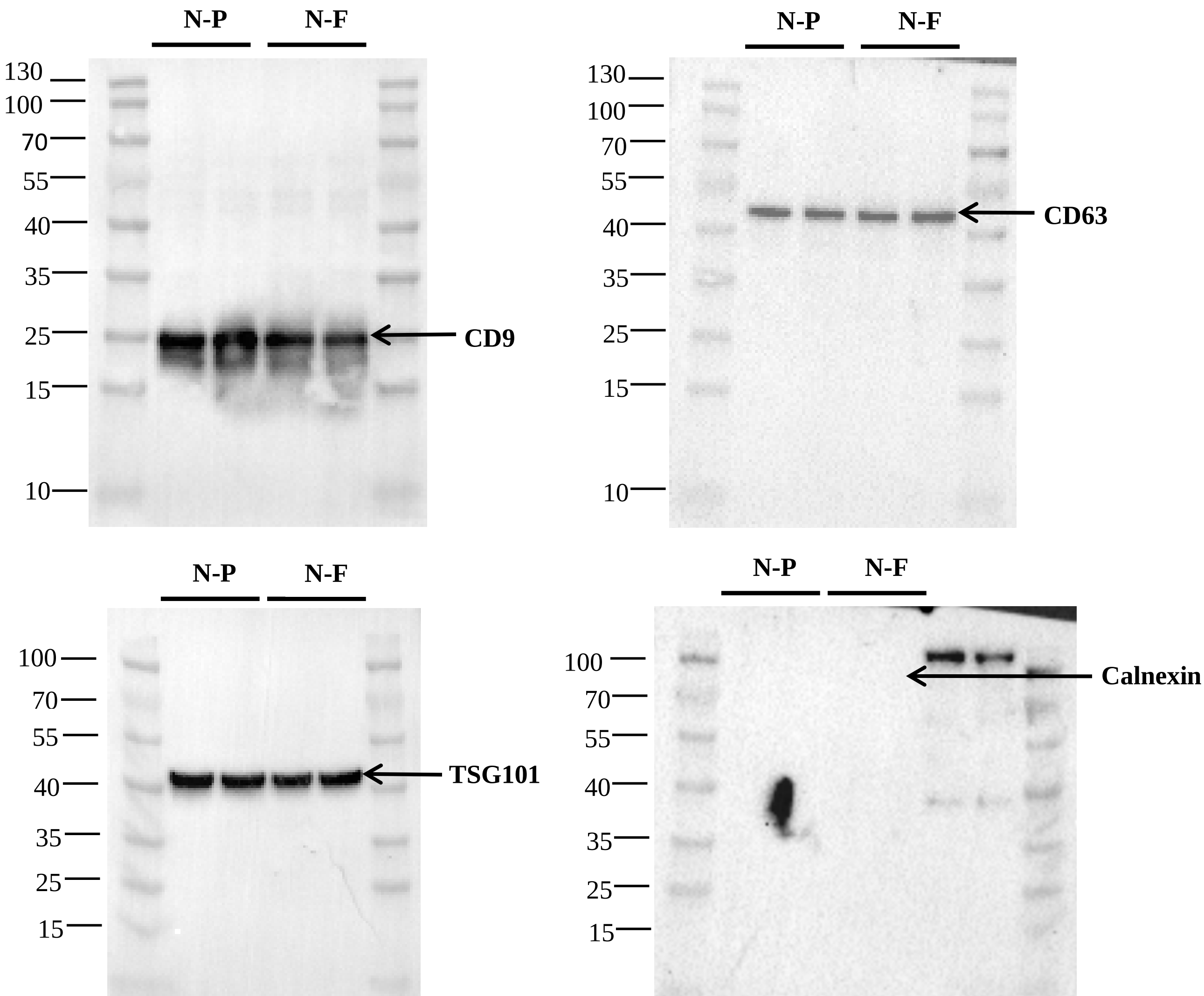

Original full-length uncropped western blots to Fig. S1B for N-P and N-F groups. Protein marker such as CD9, CD63, TSG101 and negative marker (Calnexin) were assessed by western blot in N-P and N-F groups.
