# Supplementary material for: Gestational age–associated differences in human umbilical cord mesenchymal stem cells: biological, paracrine, and neuroprotective properties
Source: Stem Cell Res Ther. 2026 Aug 12;17:414. doi: 10.1186/s13287-026-05242-8 (PMC13746429; doi:10.1186/s13287-026-05242-8)
Supplement: Supplementary file 3 [file 13287_2026_5242_MOESM7_ESM.docx]

**Supplementary Table S1.** Clinical characteristics of the UCMSCs donor samples.

| **Sample** | **Gestational age (weeks)** | **Birth weight（g）** | **Apgar score 1 min** | **Apgar score 5 min** | **Mother age** | **Umbilical cord abnormalities** | **Meconium-stained amniotic fluid** | **Placental abnormalities** | **Severe preeclampsia** | **Gestational diabetes** | **hypothyroidism**  **in pregnancy** |
| --- | --- | --- | --- | --- | --- | --- | --- | --- | --- | --- | --- |
| N-F 1 | 38^+6^ | 3530 | 10 | 10 | 35 | None | None | None | 0 | 0 | 0 |
| N-F 2 | 39^+4^ | 3680 | 10 | 10 | 30 | None | None | None | 0 | 0 | 0 |
| N-F 3 | 39^+1^ | 3670 | 10 | 10 | 31 | None | None | None | 0 | 0 | 0 |
| N-F 4 | 40^+1^ | 3280 | 10 | 10 | 37 | None | Grade I | None | 0 | 0 | 0 |
| N-F 5 | 38^+5^ | 3540 | 10 | 10 | 25 | None | None | None | 0 | 0 | 0 |
| N-P 1 | 30 | 1500 | 9 | 9 | 22 | None | None | None | 0 | 0 | 0 |
| N-P 2 | 31^+2^ | 1570 | 9 | 10 | 32 | None | None | None | 0 | 0 | 0 |
| N-P 3 | 30^+4^ | 1550 | 8 | 9 | 27 | None | None | None | 0 | 0 | 1 |
| N-P 4 | 28^+6^ | 1060 | 8 | 9 | 28 | TKUC | None | None | 0 | 0 | 0 |
| N-P 5 | 17^+2^ | 160 | NA | NA | 34 | None | None | None | 0 | 0 | 0 |
| P-GD 1 | 27^+5^ | 970 | 7 | 8 | 35 | None | None | None | 0 | 1 | 0 |
| P-GD 2 | 27^+6^ | 1030 | 7 | 7 | 30 | None | None | None | 0 | 1 | 0 |
| P-GD 3 | 28^+3^ | 1370 | 10 | 10 | 31 | None | None | None | 0 | 1 | 0 |
| P-GD 4 | 29^+4^ | 1370 | 9 | 10 | 41 | None | None | None | 0 | 1 | 1 |
| P-GD 5 | 29^+5^ | 1770 | 8 | 9 | 34 | None | None | None | 0 | 1 | 0 |
| P-SE 1 | 28^+5^ | 1080 | 7 | 8 | 34 | None | None | None | 1 | 0 | 1 |
| P-SE 2 | 31^+3^ | 1200 | 8 | 9 | 33 | None | None | None | 1 | 0 | 0 |
| P-SE 3 | 27^+6^ | 850 | 8 | 9 | 27 | None | None | None | 1 | 0 | 0 |
| P-SE 4 | 27^+6^ | 1195 | 8 | 8 | 35 | None | Grade II | None | 1 | 0 | 0 |
| P-SE 5 | 30^+3^ | 1100 | 8 | 9 | 25 | None | None | None | 1 | 0 | 0 |

Note: 0 = no; 1 = yes. **N-F**, uncomplicated full-term; **N-P**, uncomplicated preterm; **P-GD**, preterm with gestational diabetes exposure; **P-SE**, preterm with severe preeclampsia exposure; TKUC, True Knots of the Umbilical Cord

**Supplementary Fig. S1**

**Fig. S1** Characterization of EVs (A) The morphology of EVs were displayed by TEM imaging. Scale bar = 100 nm. (B) Protein marker such as CD9, CD63, TSG101 and negative marker (Calnexin) were assessed by western blot.

**Supplementary Fig. S2**

**Fig. S2** RT-qPCR validation**.** (A) Primer information (B-G) Analysis of the relative expression levels of CTNNB1, WNT5A, SIX2, SRC, ANXA2, YAP1 using reverse transcription qualitative PCR. Data are presented as mean ± SD. Statistical significance is indicated in the figure. ***p* < 0.01, ****p* < 0.001
